# Supplementary material for: Structural basis for mechanotransduction in a potassium-dependent mechanosensitive ion channel
Source: Nat Commun. 2022 Nov 12;13:6904. doi: 10.1038/s41467-022-34737-0 (PMC9653487; doi:10.1038/s41467-022-34737-0)
Supplement: Supplementary file 1 — Supplementary Information [file 41467_2022_34737_MOESM1_ESM.pdf]

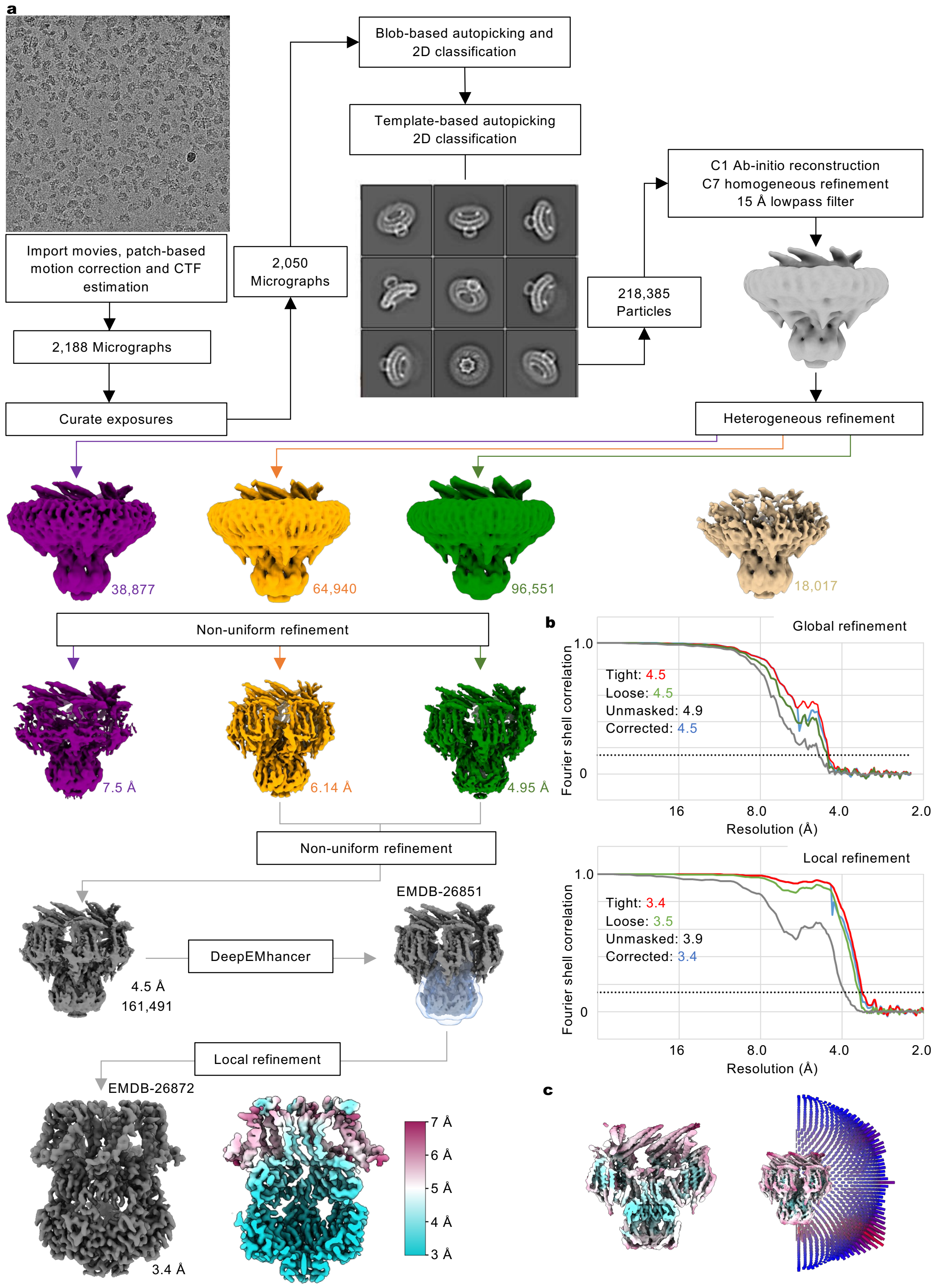


**Supplementary Fig. 1 | Cryo-EM reconstruction of the wild-type *Ec*MscK.** **a**, Flowchart of image processing in CryoSPARC v3.3. Half maps from the final round of non-uniform refinement were sharpened by DeepEMhancer using the highRes setting. Focused refinement of the channel core, including TM9-11 and the CTD, was performed within a soft mask, resulting in a higher resolution reconstruction of the core. **b**, Fourier shell correlations (FSC) of the global and local refinements. The dotted lines indicate a threshold of 0.143. **c**, Local resolution plot of the full channel and angular distribution of final particles.

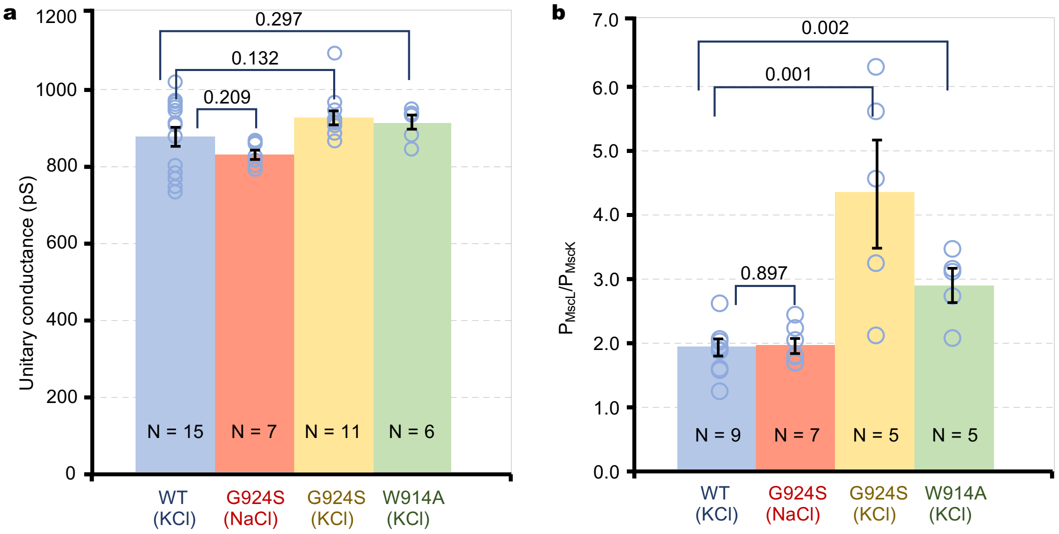


**Supplementary Fig. 2 | Gating properties of the WT *Ec*MscK and mutants. a**, Unitary conductance of the WT *Ec*MscK in KCl, G924S in NaCl or KCl, and W914 in KCl recording solutions. The numbers of independent membrane patches are indicated in the bar graphs (P values are presented for each mutant versus WT, unpaired two-tailed Student's t-test). Data are presented as mean values ± SEM. **b**, Pressure activation thresholds, measured as ratios of $P_{MscL}/P_{MscK}$, of the WT *Ec*MscK in KCl, G924S in NaCl or KCl, and W914 in KCl recording solutions. Data are presented as mean values ± SEM (P values are presented for each mutant versus WT, unpaired two-sided Student's t-test). Empty circles indicate individual data points. Source data are provided as a Source Data file.

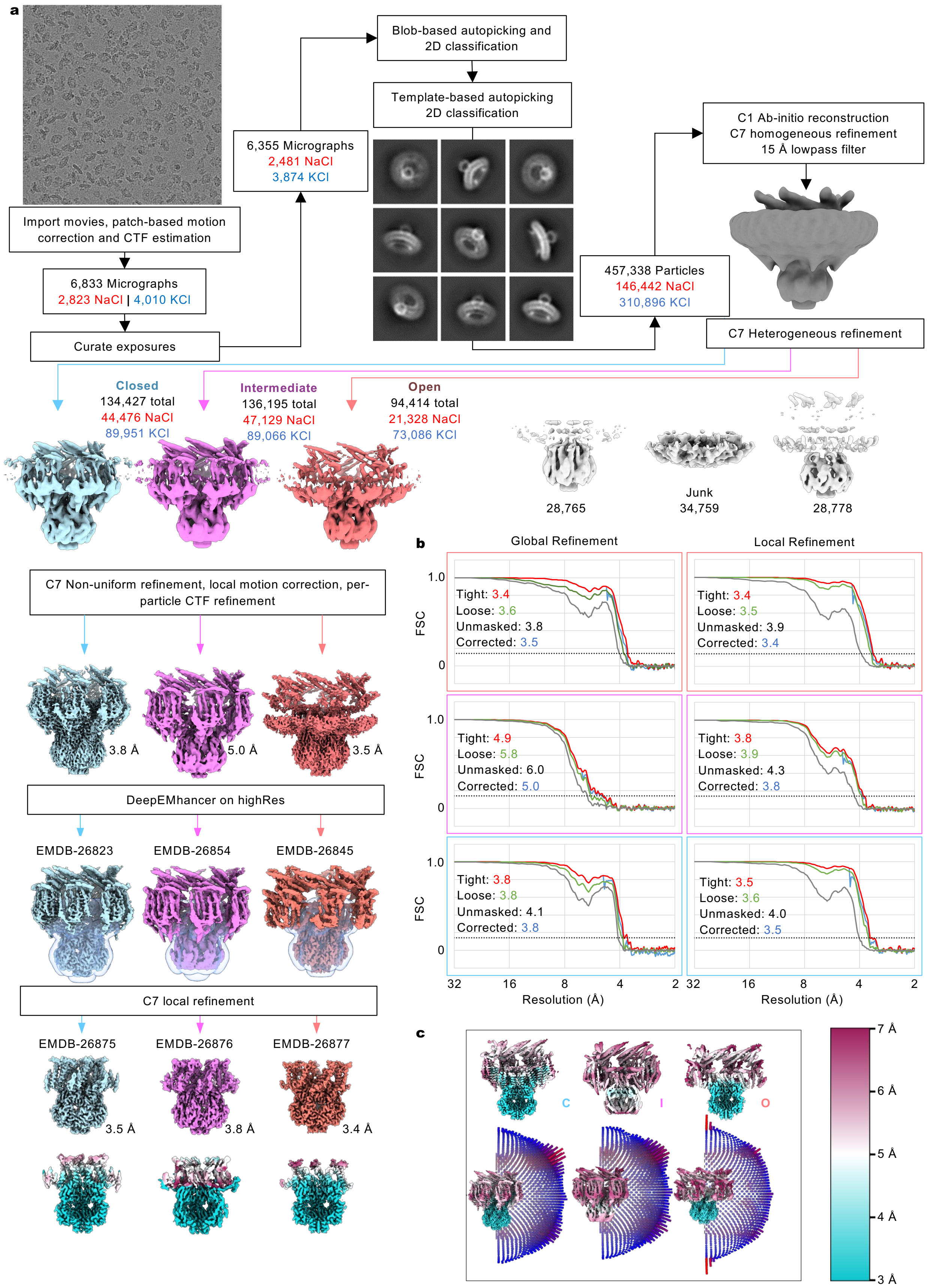
a
Import movies, patch-based motion correction and CTF estimation
6,833 Micrographs
2,823 NaCl | 4,010 KCl
Curate exposures
6,355 Micrographs
2,481 NaCl
3,874 KCl
Blob-based autopicking and 2D classification
Template-based autopicking 2D classification
457,338 Particles
146,442 NaCl
310,896 KCl
C1 Ab-initio reconstruction
C7 homogeneous refinement
15 Å lowpass filter
C7 Heterogeneous refinement
Closed
134,427 total
44,476 NaCl
89,951 KCl
Intermediate
136,195 total
47,129 NaCl
89,066 KCl
Open
94,414 total
21,328 NaCl
73,086 KCl
28,765
Junk
34,759
28,778
C7 Non-uniform refinement, local motion correction, per-particle CTF refinement
3.8 Å
5.0 Å
3.5 Å
DeepEMhancer on highRes
EMDB-26823
EMDB-26854
EMDB-26845
C7 local refinement
EMDB-26875
EMDB-26876
EMDB-26877
3.5 Å
3.8 Å
3.4 Å
b
Global Refinement
Local Refinement
FSC
1.0
0
Tight: 3.4
Loose: 3.6
Unmasked: 3.8
Corrected: 3.5
Tight: 3.4
Loose: 3.5
Unmasked: 3.9
Corrected: 3.4
Tight: 4.9
Loose: 5.8
Unmasked: 6.0
Corrected: 5.0
Tight: 3.8
Loose: 3.9
Unmasked: 4.3
Corrected: 3.8
Tight: 3.8
Loose: 3.8
Unmasked: 4.1
Corrected: 3.8
Tight: 3.5
Loose: 3.6
Unmasked: 4.0
Corrected: 3.5
32 16 8 4 2
Resolution (Å)
c
C
I
O
7 Å
6 Å
5 Å
4 Å
3 Å

**Supplementary Fig. 3 | Structure determination of *Ec*MscK G924S. a**, Flowchart of image processing of G924S in CryoSPARC v.3.3, which yielded a closed, an intermediate, and an open conformation. Micrographs collected from G924S purified in 150 mM KCl or 150 mM NaCl were combined. Half maps from the final round of non-uniform refinement were sharpened using DeepEMhancer on the highRes setting to better resolve weaker densities and interpret anisotropic features of the map. The channel core, including TM9-11 and CTD, was refined within a soft mask to improve the local resolution. **b**, Fourier shell correlations (FSC) of the final non-uniform and local refinements. The dotted lines indicate a threshold of 0.143. **c**, Angular distribution of final particles for each conformational state alongside with local resolution estimate.

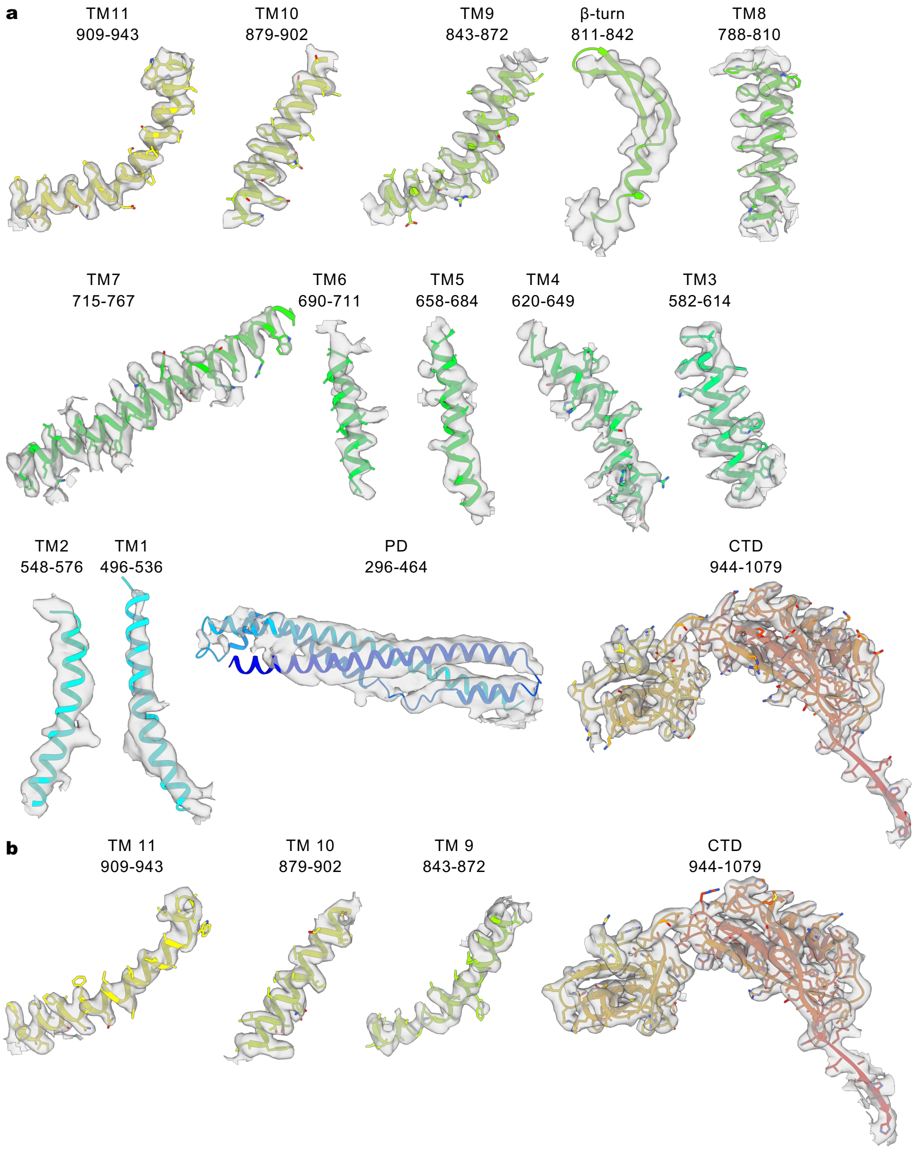


**Supplementary Fig. 4** | **Cryo-EM density.** **a**,**b**, Densities for protein segments in the closed (**a**) and open (**b**) states of G924S.

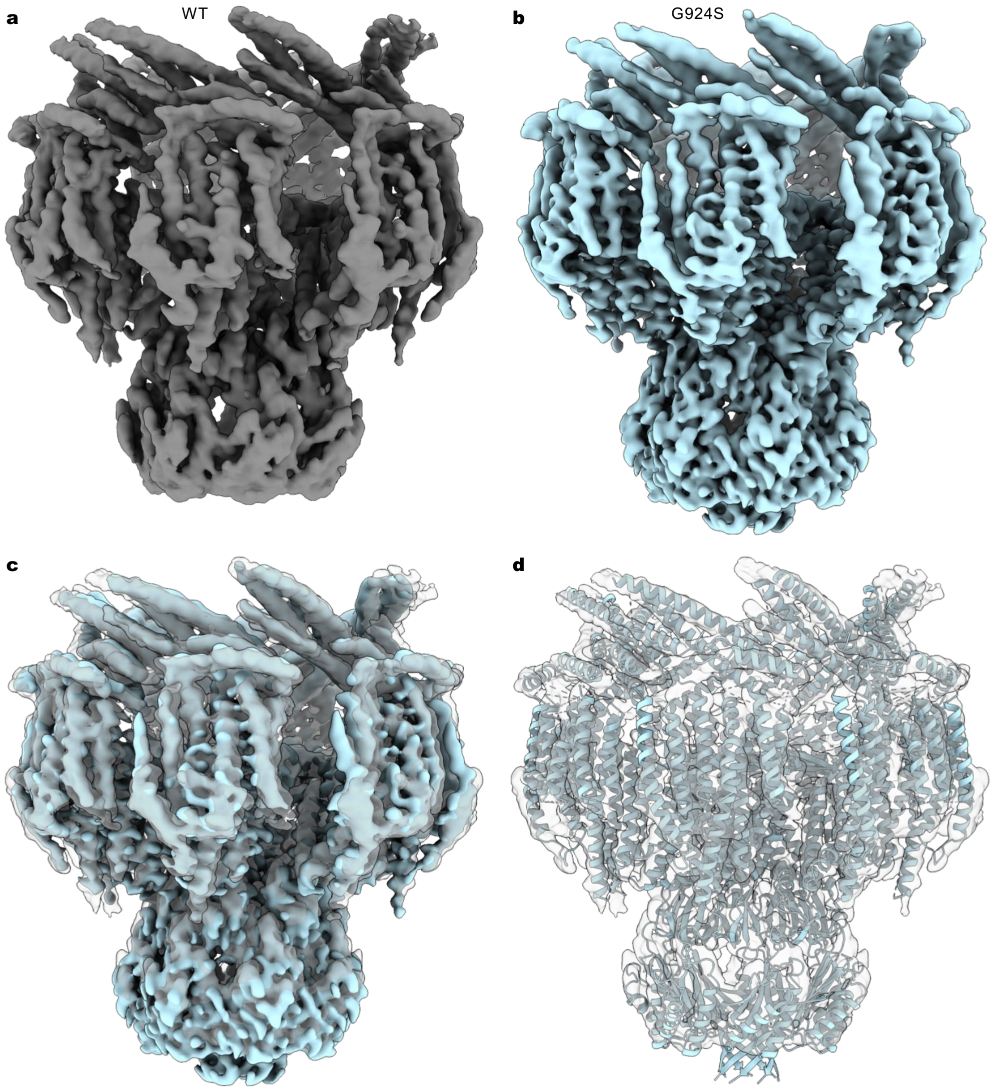


**Supplementary Fig. 5 | Comparison of cryo-EM reconstructions of the WT *Ec*MscK and G924S in the closed state. a**,**b**, Cryo-EM reconstructions of the WT *Ec*MscK (**a**) and closed *Ec*MscK G924S (**b**), which was lowpass filtered to the same resolution as that of the wild type (4.5 Å) for comparison. **c**, Overlay of the wild type and G924S reconstructions. **d**, The closed structure of G924S fits well into the cryo-EM density of the wild-type channel.

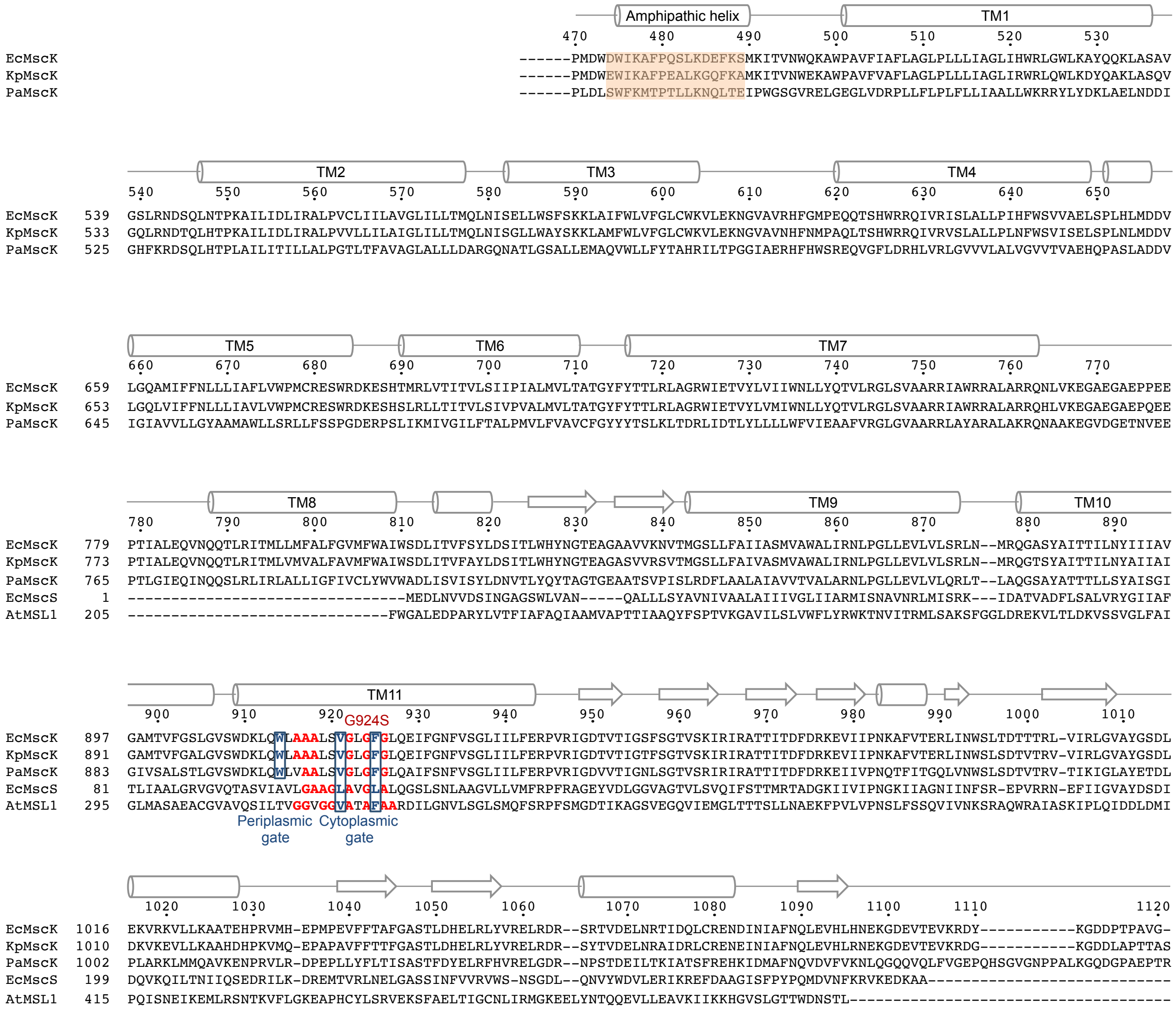


**Supplementary Fig. 6 | Sequence alignment of MscS homologs.** Aligned MscS family members include *E. coli* MscK (*Ec*MscK, NCBI sequence: NP_414998.1), *P. aeruginosa* MscK (*Pa*MscK, NCBI sequence: WP_004352503.1), *K. pneumoniae* MscK (*Kp*MscK, NCBI sequence: WP_022631229.1), *E. coli* MscS (*Ec*MscS, NCBI sequence: ATZ33519.1), and *A. thaliana* MSL1 (*At*MSL1, NCBI sequence: NP_567165.2). Secondary structure elements of *Ec*MscK are indicated. Alanine and glycine residues in the pore-lining helices are highlighted in red, and gating residues are indicated.

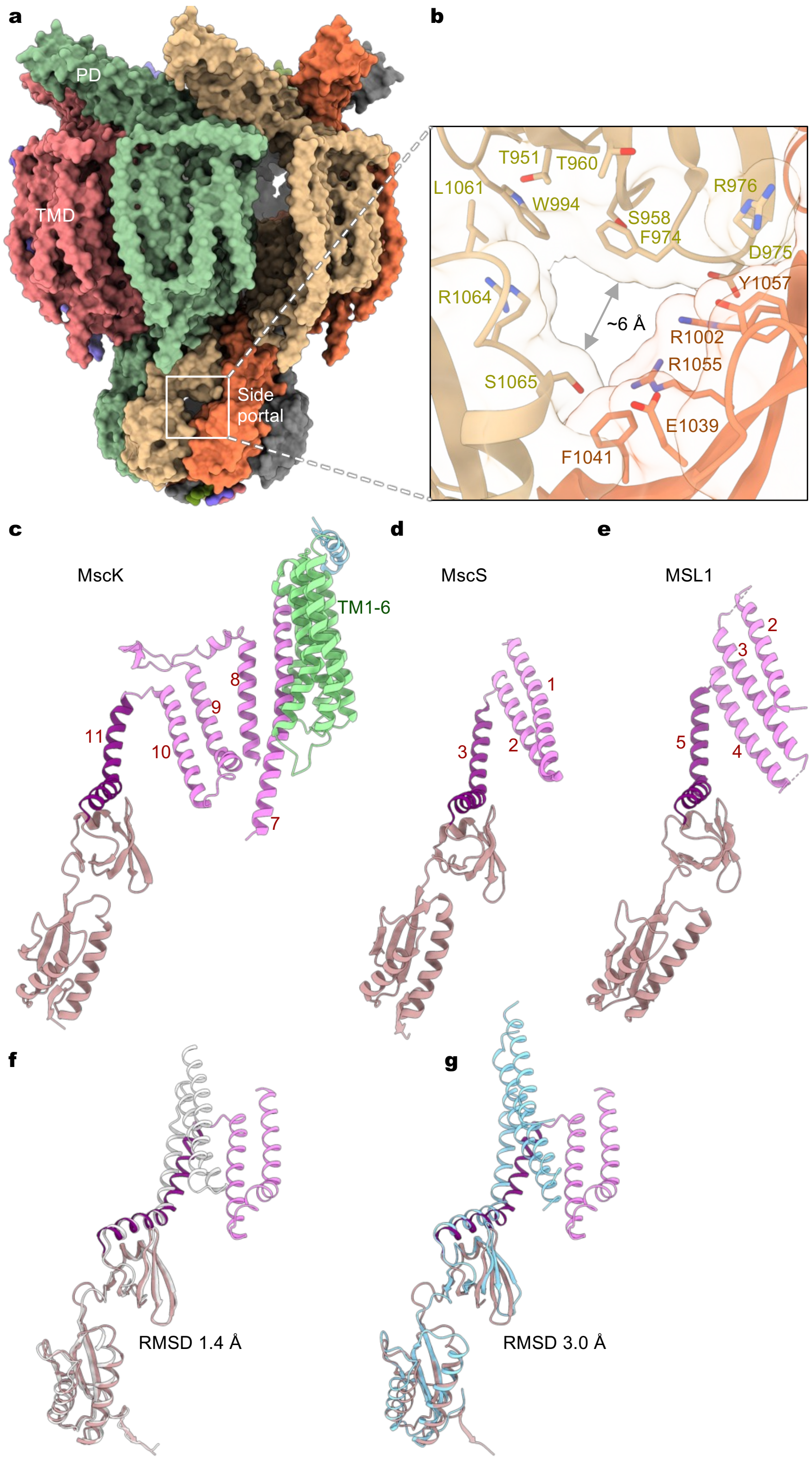


**Supplementary Fig. 7 | Structural comparison of *Ec*MscK, *Ec*MscS, and *At*MSL1. a**, *Ec*MscK and the common C-terminal side portal. **b**, Details of the side portal. **c**-**e**, Protomer structure of *Ec*MscK (**c**), *Ec*MscS (PDB: 2OAU) (**d**), and *At*MSL1 (PDB: 6VXM) (**e**). The CTD and the inner transmembrane helices are in the same colors in each structure. **f**,**g** Superpositions of *Ec*MscS (gray, **f**) and *At*MSL1 (cyan, **g**) with *Ec*MscK, respectively, by the conserved cytoplasmic CTD. The root-mean-square deviations (RMSD) of Cα for the CTDs are indicated.

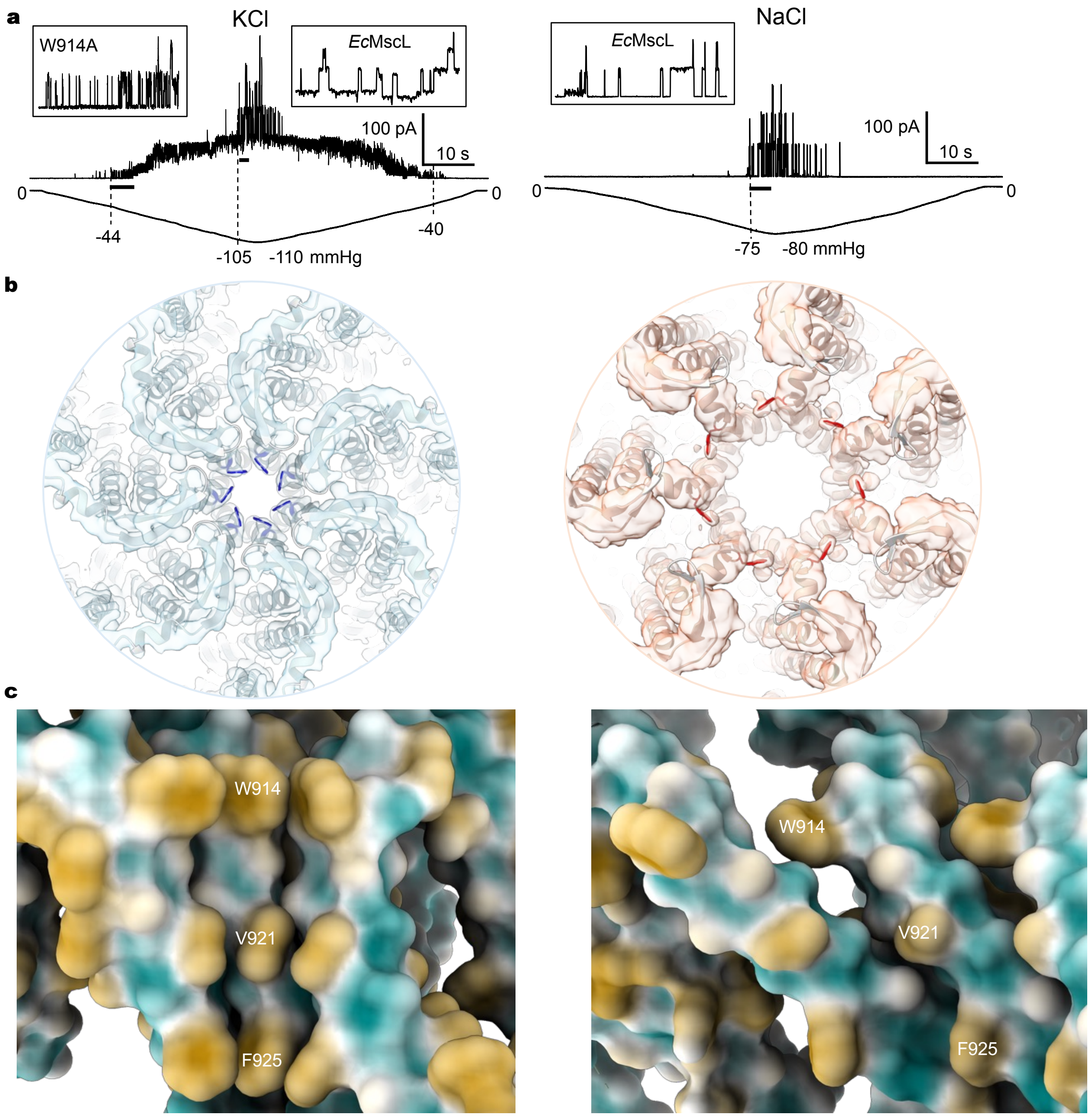


**Supplementary Fig. 8 | The role of W914 in gating. a**, Electrophysiological recording from an excised inside-out patch of *Ec*MscK W914A in symmetric KCl (left panel) or NaCl (right panel) condition. W914A opens in symmetric KCl, but not in symmetric NaCl recording conditions, and gating is characterized by rapid transitions between the open and closed states. **b**, Cross sectional views from the periplasmic side of the closed (light blue) and open (light red) conformations. **c**, W914 undergoes a significant conformational change during the gating transition from the closed (left panel) to open (right panel) state, which would be hindered by steric obstacles. The protein surface is colored by the lipophilicity potential, with yellow and blue representing hydrophobic and hydrophilic surface, respectively.

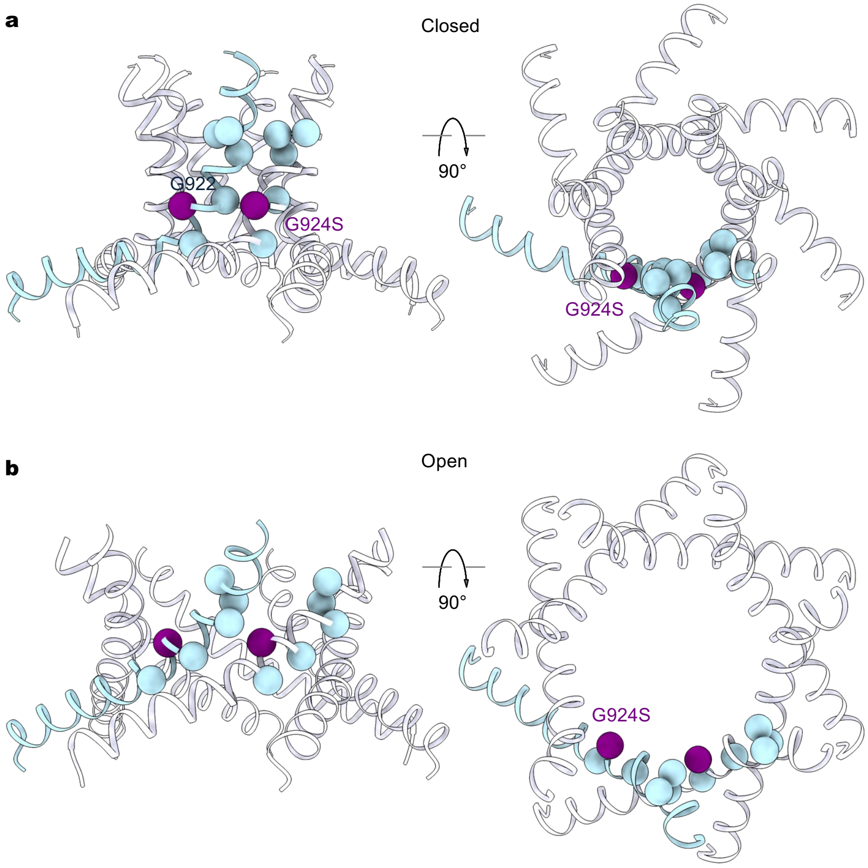


**Supplementary Fig. 9 | G924S and helix packing. a**,**b**, Packing of pore-lining helices in the closed (**a**) and open (**b**) states. Glycine and alanine residues in the pore-lining helices are highlighted as spheres. Positions of the GOF mutations (G922 and G924) are labeled.

**Supplementary Table 1 | Cryo-EM data collection, refinement and validation statistics**

| | *Ec*MscK G924S Closed (EMDB-26823) (PDB 7UW5) | *Ec*MscK G924S Open (EMDB-26845) (PDB 7UX1) | *Ec*MscK G924S Intermediate (EMDB-26854) | *Ec*MscK WT Closed (EMDB-26851) |
|---|---|---|---|---|
| **Data collection and processing** | | | | |
| Magnification | 150,000 | 150,000 | 150,000 | 105,000 |
| Voltage (kV) | 200 | 200 | 200 | 300 |
| *Electron exposure ($e^{-}/Å^2$) | 46.08, 46.24 | 46.08, 46.24 | 46.08, 46.24 | 66.0 |
| Defocus range (μm) | -0.6 to -2.4 | -0.6 to -2.4 | -0.6 to -2.4 | -1.0 to -2.5 |
| Pixel size (Å) | 0.94 | 0.94 | 0.94 | 1.10 |
| Symmetry imposed | C7 | C7 | C7 | C7 |
| Initial particle images (no.) | 457,338 | 457,338 | 457,338 | 218,385 |
| Final particle images (no.) | 134,427 | 94,414 | 136,195 | 94,247 |
| Map resolution (Å) | 3.84 | 3.47 | 4.97 | 4.5 |
| FSC threshold | 0.143 | 0.143 | 0.143 | 0.143 |
| Map resolution range (Å) | 3-6 | 3-6 | 4-7 | 3-6 |
| | | | | |
| **Refinement** | | | | |
| Initial model used (PDB code) | This Study | PDB 7UW5 | | |
| Model resolution (Å) | 4.26 | 3.78 | | |
| FSC threshold | 0.5 | 0.5 | | |
| #Map sharpening *B* factor ($Å^2$) | Variable | Variable | | |
| Model composition | | | | |
| Non-hydrogen atoms | 32,340 | 29,295 | | |
| Protein residues | 5,061 | 5,047 | | |
| Ligands | 0 | 0 | | |
| *B* factors ($Å^2$) | | | | |
| Protein | 63.05 | 116.81 | | |
| Ligand | N/A | N/A | | |
| R.m.s. deviations | | | | |
| Bond lengths (Å) | 0.005 | 0.004 | | |
| Bond angles (°) | 1.02 | 0.976 | | |
| Validation | | | | |
| MolProbity score | 1.42 | 1.50 | | |
| Clash score | 6.59 | 6.59 | | |
| Poor rotamers (%) | 0.00 | 0.00 | | |
| Ramachandran plot | | | | |
| Favored (%) | 97.75 | 97.3 | | |
| Allowed (%) | 2.25 | 2.7 | | |
| Disallowed (%) | 0.00 | 0.00 | | |

*Electron exposures are 46.08 and 46.24 $e^{-}/Å^2$ for image acquisitions in 150 mM NaCl and 150 mM KCl, respectively.
#Map sharpening was performed with DeepEMhancer.
