# Supplementary material for: Structural basis for mechanotransduction in a potassium-dependent mechanosensitive ion channel
Source: Nat Commun. 2022 Nov 12;13:6904. doi: 10.1038/s41467-022-34737-0 (PMC9653487; doi:10.1038/s41467-022-34737-0)
Supplement: Supplementary file 3 — Description of Additional Supplementary Files [file 41467_2022_34737_MOESM3_ESM.pdf]

Description of Additional Supplementary Files

File Name: Supplementary Movie 1
Description: **3DVA of the wild-type EcMscK. 3D** variability analysis shows relative motions between the CTD, TMD, and PD. Particles corresponding to the WT EcMscK were subjected to symmetry expansion in C7 and then to 3DVA at a filter resolution of 5 Å. Three major principal components were used to generate 20 intermediate states to resolve motions that account for the greatest variability. Mode 0 corresponded to relative motion of the periplasmic helix bundles, while mode 1 (shown here) and 2 corresponded to rocking of the CTD relative to the TM.

File Name: Supplementary Movie 2
Description: **Opening of EcMscK.** Morphed movie showing opening of the EcMscK channel based on the closed and open structures of the EcMscK G924S mutant. Domains are individually colored. The pore-lining helices are highlighted in magenta.

File Name: Supplementary Movie 3
Description: **Transition to the intermediate state.** Morphed movie showing transition of EcMscK from the closed to the intermediate state.
