# Supplementary material for: Structural basis for mechanotransduction in a potassium-dependent mechanosensitive ion channel
Source: Nat Commun. 2022 Nov 12;13:6904. doi: 10.1038/s41467-022-34737-0 (PMC9653487; doi:10.1038/s41467-022-34737-0)
Supplement: Supplementary file 7 — Reporting Summary [file 41467_2022_34737_MOESM7_ESM.pdf]

nature portfolio

Corresponding author(s): Peng Yuan

Last updated by author(s): Oct 26, 2022

Reporting Summary

Nature Portfolio wishes to improve the reproducibility of the work that we publish. This form provides structure for consistency and transparency in reporting. For further information on Nature Portfolio policies, see our Editorial Policies and the Editorial Policy Checklist.

Statistics

For all statistical analyses, confirm that the following items are present in the figure legend, table legend, main text, or Methods section.

n/a	Confirmed	
☐	☒	The exact sample size (*n*) for each experimental group/condition, given as a discrete number and unit of measurement
☐	☒	A statement on whether measurements were taken from distinct samples or whether the same sample was measured repeatedly
☐	☒	The statistical test(s) used AND whether they are one- or two-sided<br>*Only common tests should be described solely by name; describe more complex techniques in the Methods section.*
☐	☒	A description of all covariates tested
☒	☐	A description of any assumptions or corrections, such as tests of normality and adjustment for multiple comparisons
☐	☒	A full description of the statistical parameters including central tendency (e.g. means) or other basic estimates (e.g. regression coefficient) AND variation (e.g. standard deviation) or associated estimates of uncertainty (e.g. confidence intervals)
☐	☒	For null hypothesis testing, the test statistic (e.g. *F*, *t*, *r*) with confidence intervals, effect sizes, degrees of freedom and *P* value noted<br>*Give P values as exact values whenever suitable.*
☒	☐	For Bayesian analysis, information on the choice of priors and Markov chain Monte Carlo settings
☐	☒	For hierarchical and complex designs, identification of the appropriate level for tests and full reporting of outcomes
☒	☐	Estimates of effect sizes (e.g. Cohen's *d*, Pearson's *r*), indicating how they were calculated

Our web collection on statistics for biologists contains articles on many of the points above.

Software and code

Policy information about availability of computer code

Data collection	EPU 2 (Thermofischer Scientific), Axopatch-1D amplifier, Digidata 1320 digitizer and pClamp 8.2 software suite (Molecular Devices)
Data analysis	cryoSPARC v3.3, RELION 3.1.2, ChimeraX 1.2.5, PHENIX 1.2, COOT 0.9.6, deepEMhancer 0.13, MolProbity 4.5, pClamp 10.6 software suite (Molecular Devices), HOLE

For manuscripts utilizing custom algorithms or software that are central to the research but not yet described in published literature, software must be made available to editors and reviewers. We strongly encourage code deposition in a community repository (e.g. GitHub). See the Nature Portfolio guidelines for submitting code & software for further information.

Data

Policy information about availability of data

All manuscripts must include a data availability statement. This statement should provide the following information, where applicable:

- Accession codes, unique identifiers, or web links for publicly available datasets
- A description of any restrictions on data availability
- For clinical datasets or third party data, please ensure that the statement adheres to our policy

All data needed to evaluate the conclusions in the paper are presented in the paper or the Supplementary Materials. The cryo-EM maps have been deposited to Electron Microscopy Data Bank with accession codes EMD-26851 (WT closed), EMD-26823 (G924S closed), EMD-26845 (G924S open), EMD-26854 (G924S intermediate), EMD-26872 (WT, focused refinement), EMD-26875 (G924S closed, focused refinement), EMD-26877 (G924S open, focused refinement), and

EMD-26876 (G924S intermediate, focused refinement). Atomic coordinates have been deposited to the Protein Data Bank (PDB) with accession codes 7UW5 (G924S closed) and 7UX1 (G924S open). The source data underlying Supplementary Fig. 2 are provided as a Source Data file.

Human research participants

Policy information about studies involving human research participants and Sex and Gender in Research.

Reporting on sex and gender	N/A
Population characteristics	N/A
Recruitment	N/A
Ethics oversight	N/A

Note that full information on the approval of the study protocol must also be provided in the manuscript.

Field-specific reporting

Please select the one below that is the best fit for your research. If you are not sure, read the appropriate sections before making your selection.

☒ Life sciences ☐ Behavioural & social sciences ☐ Ecological, evolutionary & environmental sciences

For a reference copy of the document with all sections, see nature.com/documents/nr-reporting-summary-flat.pdf

Life sciences study design

All studies must disclose on these points even when the disclosure is negative.

Sample size	Electrophysiological recordings were repeated at least five times. The sample size is determined based on data consistency indicated by standard errors of the mean.
Data exclusions	For cryo-EM reconstructions of the G924S mutant channel, 457,338 particles were included for 3D classification. Three of the six classes resulted in poor protein density and were discarded (92,302). 134,427 particles were used to reconstruct the closed state, 136,195 particles were used to reconstruct the intermediate state, and 94,414 particles were used to reconstruct the open state. For cryo-EM reconstruction of the WT channel, 218,385 particles were included for 3D classification. Two of the four classes resulted in poor transmembrane density and were discarded (56,894). 161,491 particles were used in the final reconstruction for the WT channel.
Replication	Electrophysiological experiments were duplicated. Results are reproducible.
Randomization	Randomization is not applicable in cryoEM experiments. For electrophysiology, spheroplasts were randomly selected (each patched only once), and other types of randomization is not relevant in this study.
Blinding	The investigators were blinded to group allocation during data collection and analysis.

Reporting for specific materials, systems and methods

We require information from authors about some types of materials, experimental systems and methods used in many studies. Here, indicate whether each material, system or method listed is relevant to your study. If you are not sure if a list item applies to your research, read the appropriate section before selecting a response.

Materials & experimental systems

n/a	Involved in the study	
☒	☐	Antibodies
☐	☒	Eukaryotic cell lines
☒	☐	Palaeontology and archaeology
☒	☐	Animals and other organisms
☒	☐	Clinical data
☒	☐	Dual use research of concern

Methods

n/a	Involved in the study	
☒	☐	ChIP-seq
☒	☐	Flow cytometry
☒	☐	MRI-based neuroimaging

Eukaryotic cell lines

Policy information about cell lines and Sex and Gender in Research

Cell line source(s)	Pichia pastoris
Authentication	N/A
Mycoplasma contamination	Cell lines were not tested for mycoplasma contamination
Commonly misidentified lines (See ICLAC register)	N/A
